# Ectodysplasin A (EDA) Signaling: From Skin Appendage to Multiple Diseases

**DOI:** 10.3390/ijms23168911

**Published:** 2022-08-10

**Authors:** Ruihan Yang, Yilan Mei, Yuhan Jiang, Huiling Li, Ruixi Zhao, Jian Sima, Yuyuan Yao

**Affiliations:** Laboratory of Aging Neuroscience and Neuropharmacology, School of Basic Medicine and Clinical Pharmacy, China Pharmaceutical University, Nanjing 210009, China

**Keywords:** EDA signaling, skin appendage, HED, cancer, NAFLD

## Abstract

Ectodysplasin A (EDA) signaling is initially identified as morphogenic signaling regulating the formation of skin appendages including teeth, hair follicles, exocrine glands in mammals, feathers in birds and scales in fish. Gene mutation in EDA signaling causes hypohidrotic ectodermal dysplasia (HED), a congenital hereditary disease with malformation of skin appendages. Interestingly, emerging evidence suggests that EDA and its receptors can modulate the proliferation, apoptosis, differentiation and migration of cancer cells, and thus may regulate tumorigenesis and cancer progression. More recently, as a newly discovered hepatocyte factor, EDA pathway has been demonstrated to be involved in the pathogenesis of nonalcoholic fatty liver disease (NAFLD) and type II diabetes by regulating glucose and lipid metabolism. In this review, we summarize the function of EDA signaling from skin appendage development to multiple other diseases, and discuss the clinical application of recombinant EDA protein as well as other potential targets for disease intervention.

## 1. Introduction

Secreted ectodysplasin A (EDA), a distinct member of the tumor necrosis factor (TNF) superfamily, is critical for the formation of skin appendages during development [1,2]. EDA signaling is mediated by EDA, EDAR, and EDARADD, which form a unique TNF ligand–receptor–adapter protein complex mainly restricted to the skin appendages of vertebrates from fish to human [3]. In humans, mutations in any of these three genes lead to ectodermal dysplasia (ED), featured by the lacking or malformation of one or more skin appendages including hair follicles, nails, teeth and eccrine sweat glands and Meibomian glands [4]. Clinically, according to whether the patient has abnormal sweat glands, ectodermal dysplasia is divided into hypohidrotic ectodermal dysplasia (HED) and hidrotic ectodermal dysplasia [5]. HED, as the most common genetic disorder affecting ectoderm development, is characterized by dysplasia of multiple skin appendages, resulting in thinning hairs, malformed teeth, dysplasia sweat glands and impaired meibomian glands [6,7,8]. Interestingly, emerging studies have revealed the unexpected functions of the EDA pathway in multiple other diseases including various types of carcinogenesis [9,10], nonalcoholic fatty liver disease (NAFLD) [11], and androgenetic alopecia (AGA) [12]. Based on these findings, increased efforts by targeting EDA signaling have been made to interfere with these diseases. For example, the humanized recombinant EDA-A1 protein EDI200 has shown efficacy to improve HED [13], and EDAR is thought to be a promising druggable target for inhibiting carcinogenesis [9,10].

## 2. EDA Signaling

**Figure 1:**
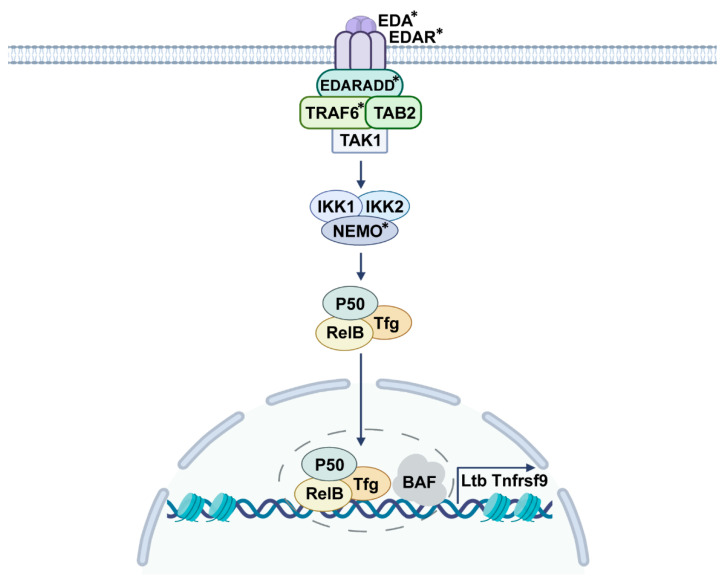
Schematic drawing of the signaling transduction of EDA pathway. Secreted EDA binds to its receptor EDAR and forms a complex with the intracellular EDARADD, then recruits TAK1, TAB2, and TRAF6, induces IKK phosphorylation, and translocates NF-κB into the nucleus, followed by attaching with the BAF complex, and eventually facilitates specific gene transcription (*: gene mutations identified in HED patients).

Secreted EDA protein has several splicing variants, of which EDA-A1 or EDA-A2 specifically binds to A1-receptor EDAR and A2-receptor X-linked ectodysplasin A receptor (XEDAR), respectively [2]. EDA-A1 is a type-II membrane protein composed of 391 amino acids, which can be cleaved by furin protease to produce EDA [1]. EDAR is a typical member of the TNF receptor family, consisting of a signal peptide, three cysteine-rich domains (CRDS), a transmembrane region, and an intracellular region containing a death domain [1]. To date, NF-κB pathway is found to be the main downstream signaling upon EDA/EDAR activation [3]. Studies have shown that the reduction of EDA signal effectively decreases NF-κB activity, and mutations in the TNF homologous domain of EDA cause the failure of EDA secretion and its retention in the cytoplasm, then repress the signaling transduction of EDA/EDAR/NF-κB pathway [14,15]. Once EDA-A1 binds to its membrane receptor EDAR, the intracellular death domain of EDAR complexes with EDARADD and recruits TAK1, TAB2, and TRAF6, leads to IκB kinase (IKK) phosphorylation, and eventually releases NF-κB to translocate into the nucleus for gene expression [16] (Figure 1). Although XEDAR lacks a death domain, it still can recruit TRAF3 and TRAF6 to activate the NF-κB pathway, as well as the JNK pathway [17]. In terms of EDA-mediated gene transcription mechanisms, our findings have demonstrated that a SWI/SNF (BAF) chromatin remodeling complex cooperates with NF-κB subunit RelB to regulate the specific gene transcription and facilitate organ development [18] (Figure 1).

EDA-mediated NF-κB activation does not appear to be associated with inflammation. However, the mutations in NF-κB-related genes such as TRAF6 not only result in symptoms of HED, but also cause inflammatory defects [19]. In particular, the gene mutations of IKKγ and IκBα have been demonstrated to cause a rare immunodeficiency ectodermal dysplasia (HED-ID), in which patients are immunocompromised and prone to persistent infection [20,21]. However, the complex crosstalk between EDA signaling and inflammatory response remains to be further investigated.

## 3. EDA Pathway and Diseases

**Figure 2:**
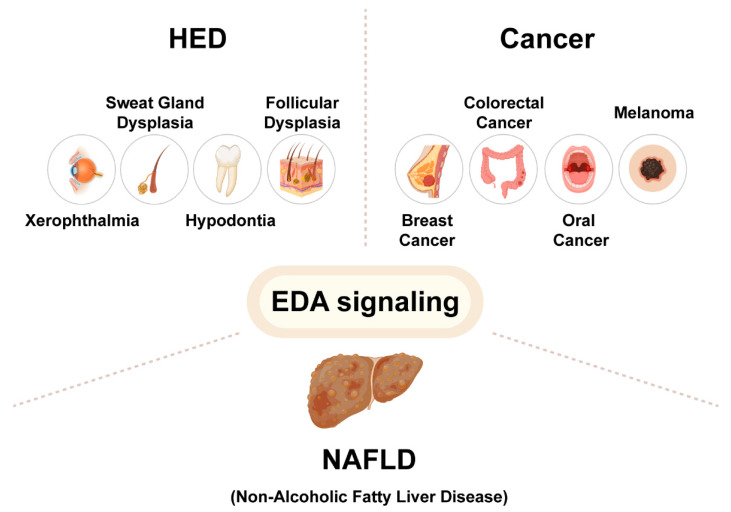
The EDA pathway involved in the pathogenesis of HED, cancer, and NAFLD. Gene mutations in the EDA pathway lead to hypohidrotic ectodermal dysplasia (HED), with clinical symptoms including xerophthalmia, sweat gland dysplasia, hypodontia, and follicular dysplasia. EDA signaling is also involved in tumorigenesis and cancer progression of breast cancer, colorectal cancer, oral cancer, and melanoma. In addition, EDA, as a newly discovered hepatocyte factor, may be highly involved in the pathogenesis of nonalcoholic fatty liver disease (NAFLD).

Evidence suggests that EDA signaling is involved in many diseases, including ectodermal dysplasia, colorectal cancer, oral cancer, melanoma, and nonalcoholic fatty liver disease (NAFLD). Based on these findings, additional components and potential targets in the EDA pathway deserve to be further studied for the possible intervention of these diseases (Figure 2).

### 3.1. Hypohidrotic Ectodermal Dysplasia (HED)

As a genetic disease, HED has several subtypes including autosomal dominant inheritance, recessive inheritance, and X-linked inheritance. Of these, X-linked HED (XL-HED) is the most common form [22]. Interestingly, XL-HED was first classified by Charles Darwin in 1875 in his book *The Variation of Plants and Animals under Domestication* [23], in which he reported the male-specific inheritance pattern of skin appendages in a family. To date, three genes including EDA, EDAR, and EDARADD mutated under different conditions are identified from patients. The X-linked EDA gene is mutated in more than 90% of cases, and the EDAR and EDARADD genes contribute to the majority of rare cases of autosomal inheritance [24,25,26]. The vast majority of XL-HED patients are male, and patients often suffer from high fever due to a lack of sweating function in infancy, which is life-threatening. In addition, Adult XL-HED patients have special facial features and may have certain defects in teeth, eyes, and ears, which significantly reduce their life quality [5]. So far, no proven treatment for XL-HED is available. Thus, accurate genetic counseling and prenatal diagnosis are necessary to guide the family to understand the risks of XL-HED [27]. Specifically, chromosome analysis of fetal amniotic fluid and sequencing analysis of mutation sites can be utilized to observe whether gene mutations occur in the EDA pathway [28]. Sparse teeth and hypoplasia of the mandible also can be observed by checking XL-HED fetuses in utero. Moreover, non-invasive prenatal ultrasonography has been recently developed to detect the average number of maxillary and mandibular teeth between 20 and 24 weeks of gestation [29,30].

Animal models of HED have been used since early in the last century. Mouse strains of Tabby, Downless, and Crinkled have been proven to be mutated in the EDA, EDAR, and EDARADD genes, respectively [16]. Confirming their roles in the EDA signaling pathway, the mutant mouse strains show similar phenotypes to HED patients in skin appendage formation, which includes a lack of sweat glands (SGs) and two types of mouse hair, “Guard” and “Zigzag” [16]. Thus, patients and mouse models have demonstrated the action of the EDA pathway, providing an ideal system to study the development of ectoderm tissues. Among the deficient skin appendages shown in Tabby mice, the formation of SGs has been mostly studied, since they are completely lacking in Tabby. As the most important skin appendage, SGs can secrete up to 1 L of sweat per day to maintain a steady body temperature [31]. It has been found that the formation of SGs is initiated by the classic Wnt/β-catenin pathway, regulated by Wnt antagonist DKK4, and subsequently controlled by the EDA and Shh pathways to proceed further development [32]. Additional molecules including FGF, BMP, Hedgehog, and other morphogen signaling pathways are also involved in the development of SGs. Among these pathways, EDA signaling is a central hub that links upstream Wnt pathway and downstream gene expression during development.

Studies have shown evidence that treatment of EDA-deficient mice with recombinant EDA protein (Fc-EDA-A1) across the placental barrier can rescue the glandular dysplasia phenotype in some offspring [33]. Fc-EDA is a recombinant fusion protein consisting of the receptor binding portion of EDA-A1 and the Fc domain of human immunoglobulin G1. In validating the XL-HED canine model, postpartum intravenous administration partially rescued the XL-HED phenotype, and prenatal ultrasound-guided intra-amniotic injection of Fc-EDA also ameliorated developmental impairment; sweating ability and meibomian gland development tend to be normal [13]. Dogs treated with FC-EDA are resistant to respiratory infections common in XL-HED conditions. Furthermore, short-term therapy has also shown promising results. For example, EDI200, based on a fully humanized FC-EDA-A1 protein, is a promising treatment for HED in humans. Although a number of neonatal clinical trials by postnatal administration of EDI200 did not provide the expected improvement in skin appendage development, in subjects given prenatal amniotic administration, SG development and sweating capacity were restored [34]. Notably, in a case of prenatal diagnosis of an XL-HED fetus, FC-EDA was injected with ultrasound-guided amniocentesis at 26 weeks of gestation, and was given it again at 31 weeks. The outcomes showed that SG and tooth development were fully recovered after birth [35]. Injecting FC-EDA directly into the amniotic fluid surrounding the EDA-deficient fetus also proved to correct some HED phenotypes, but there are still some issues need to be solved: for example, (1) Whether different mutation types and different phenotypes can use the same treatment; and (2) Different drug periods may lead to different results, and amniocentesis may have a certain risk of abortion [36]. However, based on current clinical trial data, recombinant EDA protein is still the most promising therapeutic biomedicine for HED and will hopefully be approved for clinical use in the future.

### 3.2. Cancers

EDA signaling mainly regulates skin appendage development, but emerging evidence suggests that the receptor EDAR, solely in the absence of EDA, may have unexpected functions involved in tumorigenesis and cancer progression.

EDAR, as an EDA receptor, regulates the development of mammary ducts; while EDARV370A mutations, common in Latin Americans, have been shown to affect breast anatomy and lower breast density [37]. Surprisingly, a recent study suggests that EDAR is robustly expressed in human breast cancers and its elevation induces the expression of multiple genes in the WNT pathway, which is highly correlated with breast cancer [38]. Consistently, the incidence of breast cancer is increased in transgenic mouse strains expressing a high level of EDAR [38]. Thus, EDAR, as a newly identified death receptor oncogene, may regulate breast cancer progression, possibly by interacting with the WNT pathway.

Colorectal cancer is the third most commonly diagnosed cancer and ranks as the second leading cause of cancer death worldwide with poor prognosis and high recurrence. The Cancer Genome Atlas (TCGA) database has revealed an elevated expression of EDAR in colorectal cancer tissues compared to normal tissues and this pattern was confirmed by pathological analysis of patient tissues [9]. In addition, the high expression level of EDAR may be positively correlated with the classification, pathological stage, and poor prognosis of colorectal cancer. In line with these findings, the knockdown of EDAR in colorectal cancer cell lines inhibits cell proliferation and induces apoptosis [9]. Currently, surgical treatment is the main treatment for early colorectal cancer. Since most colorectal cancer is caused by polyps, early diagnosis of colorectal cancer is an effective strategy to prevent polyps progression into cancer. Given that upregulated EDAR is involved in the occurrence and development of colorectal cancer, effective methods detecting EDAR levels in tissues of patients may provide a potential biomarker for the diagnosis of colorectal cancer.

Among various types of oral cancers, tongue squamous cell carcinoma (TSCC) is the most common one, which has a survival rate less than 50% within five years [39], mainly because that tongue tissue has rich lymphatic vessels, which has a higher chance of lymph-node metastasis [40]. A study has shown that EDARADD is associated with squamous cell carcinoma of the head and neck, and the expression of EDARADD in tissues of TSCC is higher than that in adjacent tissues. Knockout of EDARADD in TSCC cells can inhibit cell proliferation and induce cell apoptosis. EDARADD may thus be a new oncogene for TSCC. The function of EDARADD in the regulation of TSCC occurrence is possibly controlled by the downstream NF-κB pathway, which is a critical factor closely related to tumorigenesis. As a double-edged sword, NF-κB regulates immune defense, cell proliferation, and cell death, which are all crucial for cancer progression [41].

In the absence of EDA-A1 ligand, EDAR-mediated cell death requires the recruitment of EDARADD and caspase-8 activation. In an EDAR conditional knockout mouse model, data show that EDAR inhibits the progression of melanoma, which is a highly malignant tumor of melanocytes that occurs in the skin. Some studies have shown that EDAR expression is markedly repressed in cutaneous melanoma samples, suggesting that EDAR may be a negative regulator for melanoma. The authors found a remarkable reduction in EDAR expression in malignant melanoma compared with that in nearby tissues. In addition, various EDAR mutations including T167I, E254K, P409L, and V416M all significantly inhibited the pro-apoptotic activity of EDAR in cell culture. EDAR therefore appears to be an atypical TNFR that induces cell death in melanoma in a receptor-dependent manner [10]. Malignant melanoma lacks effective treatment and has a poor prognosis. Recently, targeted therapies using BRAF and MEK inhibitors are recommended for early treatment, but they have some limitations [42]. Given the pro-apoptotic function of EDAR only with no EDA-A1 binding, inhibition of the expression of EDA-A1 or its interaction with EDAR may be a new method for the treatment of melanoma. Evidence has shown that EDAR knockout mice tend to develop melanoma lesions over the first 400 days, a phenotype correlating with a lower survival rate.

The epidermal growth factor receptor (EGFR) gene is one of the most frequently mutated genes in multiple cancers, particularly in non-small cell lung adenocarcinoma [43]. Notably, Soraas and Stebbing also revealed a positive correlation between EDAR polymorphism and EGFR mutation frequencies [44], showing that the EDAR gene is possibly linked with lung cancer and may have potential as a diagnostic biomarker for this disease.

Taken together, these findings suggest that EDA/EDAR signaling is closely linked to tumorigenesis including cell proliferation, differentiation, migration, and local recurrence. However, whether EDA/EDAR plays a positive or negative effect remains controversial and needs further study. Likely, its distinct action in tumorigenesis is dependent on tumor type.

### 3.3. Nonalcoholic Fatty Liver Disease (NAFLD)

With the increasing obesity epidemic, the landscape of chronic liver disease is changing and the prevalence of NAFLD is rapidly expanding in patients with metabolic disorders [45]. NAFLD is considered as a liver manifestation of insulin resistance and metabolic syndrome which alters the secretion of multiple hepatocellular cytokines [46]. EDA is a newly identified liver cytokine that can be secreted into the circulatory system to regulate energy and glycolipid metabolism [11,47]. Recent findings show that the levels of EDA in the livers of db/db mice are upregulated, suggesting EDA as a possible regulator for type-II diabetes [48]. In addition, hepatic steatosis may promote EDA expression through peroxisome proliferators-activated receptor γ (PPARγ), thereby reducing systemic insulin sensitivity [48]. Interestingly, the imbalance of EDA expression in the liver, especially the EDA-A2 subtype, is increased in hepatic steatosis, which can lead to the deterioration of insulin sensitivity and glucose homeostasis in muscle. Furthermore, the serum level of EDA-A2 in NAFLD patients is increased and positively correlated with blood glucose balance and inflammation [47].

In a hospital-based case-control study, higher serum EDA concentrations are found in patients with NAFLD and elevated EDA-A2 is associated with higher incidence of NAFLD. EDA-A2 thus could be a potential biomarker of non-invasive diagnosis of NAFLD [47], while another study shows that EDA levels in liver are higher in non-alcoholic steatohepatitis (NASH) group than in those without NAFLD; but EDA levels in NAFL and non-NAFLD patients are similar. In comparison to patients without NAFLD, plasma EDA in both the NAFLD and NASH groups are upregulated and positively correlated with the degree of steatosis [11]. These data suggest that plasma EDA may not be a reliable biomarker for NAFLD since it cannot distinguish between NAFL and NASH. The contradictory results can be caused by several reasons. First, the proportion of EDA-A2 in total EDA is unclear and EDA-A2 has been reported to have distinct functions compared to EDA-A1. Second, the diagnostic criteria for NAFLD used in each study are different [11,47]. In sum, it is deserved to verify whether circulating EDA level can be used to predict the presence of NAFLD.

### 3.4. Other Diseases

The EDA pathway also affects several other diseases. Evidence has shown that a defective EDA pathway leads to dry eye symptoms due to dysplasia of the lacrimal gland and Meibomian gland (MG) [8,49]. The EDA pathway is widely active in the basal epithelium during lacrimal gland development. EDA^−/−^ mice have lower basal tear secretion, but embryonic lacrimal gland morphology does not seem to be affected [49]. In line with this study, data have shown adult EDA^−/−^ mice have impaired cell terminal differentiation and delayed corneal wound healing [50]. Previous findings from our study have also demonstrated that EDA-deficient Tabby mice have a severe dry eye condition due to the loss of MGs [8]. Unlike its function in the lacrimal gland, the EDA pathway mainly regulates the early development of MGs [8].

Although EDA-A1/EDAR functions in hair follicle formation at early stage of skin appendage development, EDA-A2/XEDAR signaling, however, acts in hair circulation after birth. As shown by recent studies, the XEDAR gene is found to be linked to androgenetic alopecia (AGA), a common progressive hair loss disorder [51,52]. Further findings have shown that EDA-A2/XEDAR induces apoptosis in cultured human hair follicle (HF) cells and promotes the regression of HFs in mice [12]. Given the inhibitory function of EDA-A2/EDA2R signaling in hair growth, disrupting EDA-A2/EDA2R signaling could be a promising strategy for the treatment and prevention of AGA.

## 4. Perspectives

EDA signaling is involved in the regulation of various diseases, such as HED, cancers, NAFLD, type II diabetes, dry eye condition, and so on. This review highlights the roles of EDA signaling in different physiological and pathological processes, and discusses the clinical application of Fc-EDA protein in HED and the possible drug development of EDAR inhibitors for cancer treatment. However, fundamental studies of EDA signaling are still needed, specifically on its molecular mechanism and distinct roles in numerous diseases. The aspects of these efforts may include: (1) Besides the co-operation of NF-κB and BAF chromatin remodeling complex, additional transcriptional machinery regulating EDA-mediated gene expression could be investigated. (2) The distinct function of EDA-A1/EDAR and EDA-A2/XEDAR in various diseases remains to be further determined. (3) The identification of potential therapeutic targets in the EDA signaling pathway is desperately needed.

## Data Availability

Not applicable.
